# Three-dimensional Optical Coherence Tomography Imaging and Treatment of Glaucomatous Optic Nerve Head Defects Associated with Schisis-like Maculopathy

**DOI:** 10.4274/tjo.68815

**Published:** 2017-04-01

**Authors:** Zafer Öztaş, Jale Menteş, Halil Ateş, Serhad Nalçacı

**Affiliations:** 1 Ege University Faculty of Medicine, Department of Ophthalmology, İzmir, Turkey

**Keywords:** glaucoma, retinoschisis, optical coherence tomography

## Abstract

We present the three-dimensional (3D) spectral-domain optical coherence tomography (SD-OCT) findings of schisis-like maculopathy associated with structural changes of the optic nerve (ON) head as well as the treatment outcomes of a case of advanced glaucoma. In addition to ophthalmological examination, B-scan and 3D-SD-OCT images of the ON head, peripapillary retina, and the macula were obtained. The B-scan images only detected typical retinoschisis findings. However, the 3D-SD-OCT images of the ON head revealed defects of various sizes, shapes, and depths at the outer wall of the prelaminar and laminar regions of the ON canal. The 3D images were able to establish that these defects were both adjacent to and interconnected with the retinal layers. The patient successfully received 3D-SD-OCT-guided thermal laser treatment that is used in congenital optic disc pits complicated with macular schisis. In brief, 3D-SD-OCT is very useful for demonstrating the ON head defects that can lead to schisis-like maculopathy in cases of advanced glaucoma.

## INTRODUCTION

Acquired optic disc pits, a finding specific to glaucomatous optic nerve (ON) head damage, develop in association with localized depressions in the lamina cribrosa (LC). This special condition was first described by Radius et al.^1^ in 1978. Acquired optic disc pit typically appears as a pale area at the inner periphery of the optical disc margin. Unlike congenital optic disc pits, acquired pits may form as a result of neuroretinal rim loss secondary to glaucoma.

Macular retinoschisis (or schisis-like maculopathy), a complication of congenital optic disc pits, and subsequent serous retinal detachment may also develop in acquired optic disc pits and result in serious vision loss. In fact, even in the absence of a detectable pit lesion, peripapillary and macular retinoschisis and associated serous macular detachment have been reported in glaucomatous eyes with severe optic disc cupping.^2,3,4,5^ In recent years, optical coherence tomography (OCT) imaging techniques have allowed the detection of these pathologies, which can now be visualized in the peripapillary area, ON head, and even the LC with the use of advanced techniques.^6,7,8^

This study presents the three-dimensional (3D) spectral domain (SD) OCT features and treatment outcomes of prelaminar and laminar defects in the walls of the ON canal caused by advanced glaucomatous ON head damage and leading to macular retinoschisis.

## CASE REPORT

A 30-year-old man whose vision level exhibited diurnal fluctuations was referred to our clinic for bilateral macular retinoschisis. The patient underwent a detailed ophthalmologic examination including best corrected visual acuity (BCVA), slit-lamp examination, intraocular pressure measurement, fundus photography, and fluorescein angiography.

In addition, B-scan and 3D SD-OCT (Topcon 3D OCT-2000, Tokyo, Japan) images of the ON head, peripapillary retina, and macula were obtained and analyzed.

**Figure 1:**
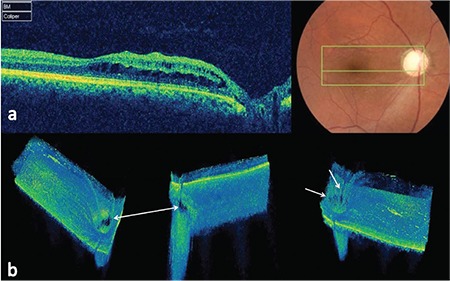
a) B-scan spectral-domain optical coherence tomography images showing retinoschisis-like cystic structures in the papillomacular and macular areas and separation of the outer retinal layers; b) three-dimensional-spectral-domain optical coherence tomography showing focal defects in the side walls of the optic nerve canal (white arrows)

The patient’s history revealed he had been diagnosed with closed-angle glaucoma and undergone bilateral trabeculectomy 6 years earlier. His BCVA fluctuated periodically, and was measured as 2/10 in the right eye and 10/10 in the left eye. Substantial BCVA fluctuations between 1/10 and 4/10 were noted in the patient’s right eye prior to treatment. The patient also reported BCVA fluctuations in his left eye; the lowest measurement we obtained was 9/10. On slit-lamp examination, the anterior chambers of both eyes appeared quiet and the surgical iridectomies were open. The corneas and crystalline lenses of both eyes were clear. Right and left intraocular pressures were 12 and 13 mmHg with latanoprost and were measured as 11 and 15 mmHg during follow-up. Fundoscopic examination revealed cup-to-disc ratios of 1.0 and 0.9 in the right and left eyes, respectively, and no abnormalities were detected other than advanced glaucomatous cupping of the optic discs. Fundus fluorescein angiography was normal in both eyes. The OCT images were examined in detail in light of these findings. B-scan SD-OCT images showed retinoschisis-like cystic structures in the papillomacular and macular areas (an area about 4x4 mm between the ON and fovea) and separation of the outer retinal layers (Figure 1a). Furthermore, to determine the underlying pathologic causes, 3B-SD-OCT images were examined and revealed focal defects of at the outer ON margins in the prelaminar and laminar regions of the ON head (Figure 1b).

**Figure 2:**
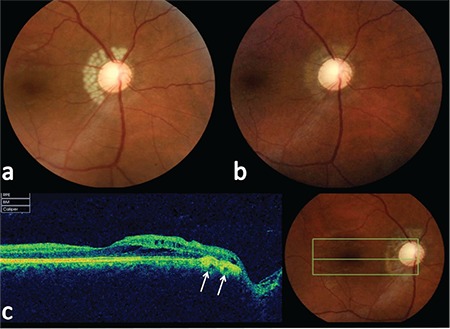
a) Peripapillary thermal laser therapy, b) peripapillary atrophy, c) B-scan optical coherence tomography showing peripapillary fibrosis and cicatrization (white arrows)

Presuming that the intraretinal fluid was probably vitreous and may have leaked between the retinal layers through these defects, the patient was treated with thermal laser photocoagulation to the peripapillary areas adjacent to the defects. Under 3D-SD-OCT guidance, thermal laser settings similar to treat congenital optic disc pits (100 μm spot size, 0.3 s duration, 200 mW power) were used to produce the desired level of whiteness to the desired area using consecutive burns (Figure 2).

**Figure 3:**
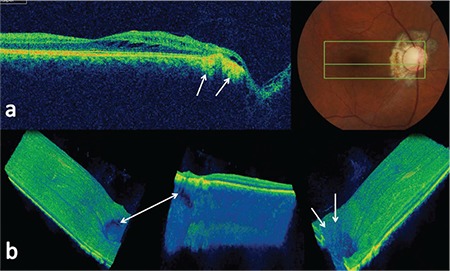
a) B-scan optical coherence tomography images taken 6 months after treatment showing marked regression of intraretinal fluid, and peripapillary fibrosis and cicatrization (white arrows); b) three-dimensional spectral-domain optical coherence tomography images showing incomplete closure of a large laminar defect (white arrow) and complete closure of small prelaminar defects (white arrows)

Within 6 months after the laser therapy induced fibrosis and cicatrization of the peripapillary areas, the focal defects in the ON periphery were observed to markedly decrease in size, though some large defects did not resolve completely. The intraretinal fluid regressed substantially and BCVA stabilized (Figure 3). Furthermore, the fluctuations in visual acuity resolved. The patient was followed for a total of 24 months, during which the favorable outcomes persisted.

Although all examinations, imaging, and treatments were applied to both eyes, the findings were more pronounced in the right eye and these images are presented in this report. Due to inconsistencies in the patient’s visual field test, evaluations related to visual field are not presented here.

## DISCUSSION

Macular retinoschisis, a term used to describe large separations of the retinal layers in the posterior pole, often accompanies congenital optic disc pits. Macular retinoschisis may also form due to various reasons such as tractional causes, venous occlusive diseases, cystoid macular edema, and juvenile retinoschisis.^9^ This condition, also called optic disc maculopathy, may also be seen with acquired optic disc pits. Cases of macular retinoschisis without a visible pit have been reported recently in patients with advanced closed- or narrow-angle glaucoma.^2^,^3^,^4^,^5^ These studies reported that retinoschisis and possibly subsequent serous macular detachment may have developed as a result of a small hole associated with elevated intraocular pressure. The aim of this case report was to investigate the 3D-SD-OCT characteristics of peripheral ON head focal defects which developed in the absence of a visible pit and led to macular retinoschisis in a patient with advanced glaucoma, and to present the results of 3D-SD-OCT-guided thermal laser therapy.

In the present study, we closely evaluated optic disc, peripapillary, and macular OCT images in order to determine the primary cause of retinoschisis in our patient. While peripapillary and macular cystoid cavities and detachment of the outer retinal layers were detected on standard B-scan SD-OCT imaging, 3D-SD-OCT imaging revealed optically dark (empty) defects in the optic disc margins at the prelaminar and laminar levels. In our search of the literature to identify these defects, we found reports of pathologies with similar appearance in recent studies using enhanced-depth imaging (EDI) OCT and swept source (SS) OCT.^6,7,8^ Using EDI-OCT, You et al.^7^ described LC holes and defects appearing as LC degeneration adjacent to the side walls of the ON canal in patients with advanced glaucomatous neuroretinal rim thinning. Takayama et al.^8^ used SS-OCT to demonstrate similar defects in the LC region in glaucomatous eyes. Kiumehr et al.^10^ examined the LC region of normal and glaucomatous eyes using EDI-OCT and found focal LC defects in 76% of glaucomatous eyes. They noted that a large proportion of these defects are not detected during routine examinations, and found that the LC of healthy eyes was intact. These advances in OCT imaging techniques have enabled the visualization of the optic disc pit and deeper optic disc structures such as the LC. These studies confirmed that focal defects may develop in the LC.^6,7,8,10^ In our patient, the focal glaucomatous defects in the peripheral ON and their relationship to the areas of macular retinoschisis were shown with 3D-SD-OCT. Due to the inadequate tissue penetration of SD-OCT, the LC area adjacent to these defects could not be clearly evaluated.

There is a debate as to whether the intraretinal fluid seen in congenital optic disc pits is cerebrospinal fluid or vitreous. However, we believe that this fluid is vitreous that leaks in through focal defects in the outer layers of the optic nerve. Nevertheless, we found no OCT findings directly supporting the vitreous as the source of this fluid.

In our patient, macular retinoschisis caused fluctuations in vision, especially in the right eye. In order to stabilize visual acuity, obtain a dry macula, and prevent possible serous macular detachment, we treated both of our patient’s eyes with thermal laser therapy as used in congenital optic disc pits, under 3D-SD-OCT guidance. Due to the lack of serous retinal detachment accompanying the macular retinoschisis and the advanced glaucomatous ON damage, pars plana vitrectomy was considered risky in this patient, whose clinical signs were relatively mild.

Following treatment, the defects in the peripheral optic cup had substantially decreased in size, but larger and deeper defects did not close completely. Green argon thermal laser therapy resulted in fibrosis and scar tissue in the peripapillary regions adjacent to the defects which could prevent leakage of the vitreous between the retinal layers. Our patient showed substantial regression of the intraretinal fluid and stabilized visual acuity after treatment.

## CONCLUSION

In summary, 3D-SD-OCT imaging is useful and effective in detecting peripheral ON defects at the prelaminar and laminar levels which result in macular retinoschisis in advanced glaucoma patients, and in determining the efficacy of treatment.
